# Sleeping mattress determinants and evaluation: a biomechanical review and critique

**DOI:** 10.7717/peerj.6364

**Published:** 2019-01-25

**Authors:** Duo Wai-Chi Wong, Yan Wang, Jin Lin, Qitao Tan, Tony Lin-Wei Chen, Ming Zhang

**Affiliations:** 1 Hong Kong Polytechnic University Shenzhen Research Institute, Shenzhen, China; 2 Department of Biomedical Engineering, Hong Kong Polytechnic University, Hong Kong, China; 3 Product Development–R&D Life Nurturing Products, Infinitus (China) Company Limited, Guangzhou, China

**Keywords:** Sleep support, Body pressure, Spine alignment, Mattress, Pillow

## Abstract

**Background:**

Sleeping mattress parameters significantly influence sleeping comfort and health, as reflected by the extensive investigations of sleeping support biomechanics to prevent sleep-related musculoskeletal problems.

**Methodology:**

Herein, we review the current trends, research methodologies, and determinants of mattress biomechanics research, summarizing evidence published since 2008. In particular, we scrutinize 18 articles dealing with the development of new designs, recommendation criteria, instruments/methods of spine alignment evaluation, and comparative evaluation of different designs.

**Results:**

The review demonstrated that mattress designs have strived for customization, regional features, and real-time active control to adapt to the biomechanical features of different body builds and postures. However, the suggested threshold or target values for desirable spine alignment and body pressure distribution during sleep cannot yet be justified in view of the lack of sufficient evidence.

**Conclusions:**

It is necessary to formulate standard objectives and protocols for carrying out mattress evaluation.

## Introduction

Some of the most frequent postures and locomotions in our lifetime are those adopted during sleeping. Therefore, sleeping systems and supports are considered to be important environmental components affecting physical comfort during sleep and thus influencing health. However, sleep disorders and problems are common, resulting in poor work efficiency, absenteeism, and accidents (Swanson et al., 2011). Additionally, poor sleep quality can cause musculoskeletal problems, including chronic pain, low back pain, and arm-shoulder pain (Cimmino, Ferrone & Cutolo, 2011; Mork et al., 2013).

Although the relationship between biomechanics and sleep-associated musculoskeletal problems has been recognized, most mattress evaluations pertain to the use of insecure techniques such as subjective feedback or questionnaires (Tonetti, Martoni & Natale, 2011; Verhaert et al., 2011b). The subjective responses gathered from questionnaires may change after an adaptation period (Liu, Lee & Liang, 2011), and the outcome of subjective comfort evaluation is argued to be easily manipulable based on manufacturer demands because of the highly diverse and vague definition of “comfort” (Wu, Yuan & Li, 2018). To introduce more order into this field, the European standardization body published the EN 1957 standard as a guide to mattress firmness selection according to hardness and area under the deflection curve (European Committee for Standardization, 2000). However, the corresponding recommendations are based on subjective perception and supine posture only (López-Torres et al., 2008) and lack supporting experimental data.

Besides subjective feedback, sleep therapists identified spine and pressure point positions as additional decisive factors for prescribing sleeping support (Esquirol Caussa et al., 2017). In general, an ideal sleeping support system should maintain the spine physiologically aligned by providing adequate and appropriate mechanical support (DeVocht et al., 2006; Verhaert et al., 2011b). In contrast, the use of sagging supports is believed to result in discomfort, poor sleeping quality, and waking symptoms caused by the irritation of adjacent soft tissue and nerves of the distorted spine (Verhaert et al., 2011b). Moreover, the body-mattress interfacial contact–pressure relationship, which is recognized as an indicator of tactile comfort, should also be taken into account (Pearson, 2009), for example, the use of rigid surfaces results in high and concentrated pressure, which induces discomfort and poor localized blood supply and can therefore place bed-borne patients at risk of pressure ulcer formation (Berlowitz & Brienza, 2007).

Although sleeping mattress biomechanics have not been extensively studied, the corresponding works employed broadly variable investigation methods and determinants (Radwan et al., 2015). Herein, we aim to review and summarize the biomechanical methods and determinants used to evaluate domestic mattress design and selection criteria and thus hope to stimulate the formalization of objective standards for the biomechanical evaluation of sleeping supports.

## Survey Methodology

### Search strategy

Article search was conducted using “sleeping,” “mattress,” and “biomechan*” as keywords. Several databases were accessed, including Medline, Pubmed, ScienceDirect, Cochrane Collaboration, Scopus, and the Web of Science. A gray literature search was conducted using Google Scholar. Studies conducted in 2008–2018 and published in peer-reviewed journals in English were considered.

### Study selection

Article relevancy was determined through primary title-based screening by two independent reviewers, and a second abstract- and full text-based screening was conducted by the same reviewers. In case of discrepancy, two more reviewers were intervened, and the selection outcome was settled by discussion. Articles were excluded if they did not account for any biomechanical measurements or investigations, for example, some excluded articles only dealt with posture behavior, cardiovascular and pulmonary measurements, questionnaires, temperature, and humidity. Articles on medical-use mattresses, particularly those describing anti-pressure sore mattresses, were also excluded.

### Data extraction

Details of the 18 articles qualified for this review are summarized in Table 1 (selection of population and mattress samples), Table 2 (methods and biomechanical outcome measures), and Table 3 (study design and research scope).

**Table 1:** Selection of subjects and mattress samples in the reviewed articles.

Author (Year)	Subjects	Exclusion criteria	Sleeping posture	Mattress characteristics	Manufacturer
Chen et al. (2014)	16 healthy males aged 20–45	Sleep disorders, vital signs beyond the normal range	Supine Lateral	Plank springs With supporting layer and pillow top made of palm fiber 3D structure made of foam rubber and plant fiber, with supporting layer, intermediate layer finely fitting the shape of the human body, and pillow top Independent springs	(1)–(4) DaZiRan Science and Technology Ltd., Guizhou, China
Denninger, Martel & Rancourt (2011)	Three subjects (1F/2M) with NS age	NS	Lateral	Custom-made mattress consisted of rows and columns of PU foam (extra-firm Q41) cubes with hollow ellipsoidal cavities. Cube dimensions were customized according to spinal curvature and body weight portion	NA
Deun et al. (2012)	11 healthy subjects (5F/6M) aged 20–28, mean age = 21.2 ± 3.2	Medical conditions interfering with normal sleep, back pain, intake of sleep medications	No control, postures were detected and estimated	Dynasleep, mattress equipped with indentation sensors and adaptive actuator spring pockets Actuator inactive Actuator active, induced different stiffness in eight zones to optimize spinal curvature based on the results of indentation measurements	Custom8, Leuven, Belgium
Esquirol Caussa et al. (2017)	First pilot test: six subjects, age/gender NS; Second pilot test: 50 subjects (28F/22M) aged 18–93, mean = 34.2; Final study: 151 subjects (60F/91M) aged 4–94, mean = 34.43; Re-analysis study: 117 subjects (75F/42M), aged 4–93, mean = 33.82	NS	Supine	Five types of mattresses (DORMITY^®^): Soft, density = 2.75 kPa* Neutral/soft, density = 3.0 kPa Neutral, density = 3.3 kPa Neutral/hard, density = 3.8 kPa Hard, density = 4.4 kPa Three types of toppers (DORMITY^®^): Soft, density = 1.1 kPa Medium, density = 1.6 kPa Hard, density = 2.1 kPa Three types of pillows of different densities (45 combinations)	Dormity.com, Barcelona, Spain
Lee et al. (2015)	10 healthy subjects (5F/5M), age mean = 29.1 ± 3.2	Any skin or musculoskeletal disorders affecting supine position;pain in the measuring site	Supine	Subjects’ existing mattress	NA
Lee et al. (2016)	10 healthy subjects (5F/5M), age mean = 29.1 ± 3.2	Any skin or musculoskeletal disorders affecting supine position;pain in the measuring site	Supine	Floor Mattress	NA NS
Leilnahari et al. (2011)	25 male students, age: NS	Spinal deformities	Lateral	Soft mattress (polyurethane foam and a layer of memory foam Firm mattress Custom-made mattress with different regional stiffness based on neutral spine alignment predicted by the musculoskeletal model. The mattress was made of a combination of PU and spiral pressure springs with different wire diameters	NS NS NA
López-Torres et al. (2008)	19 young subjects (9F/10M), age mean = 28 ± 3 (F); 26 ± 3 (M), 56 elderly subjects (34F/22M), age mean = 67 ± 5 (F); 70 ± 6 (M)	NS	A three-step testing procedure: seated position supine roll onto one side	Four mattresses were selected from 17 samples to cover the full range of firmness	NS
Low et al. (2017)	20 young healthy subjects (10F/10M), age: NS	Back, shoulder or neck pain in the past month	Supine Lateral Prone	Delight, latex foam mattress Masterfoam 1000, high-density PU foam mattress	Sofzsleep, Singapore Masterfoam, Darul Ehsan, Malaysia
Palmero et al. (2017)	200 subjects (128F/72M) aged 4–93, mean = 33.82 ± 23.02	NS	Supine	Intermediate density mattress	NS
Park, Kim & Kim (2009)	64 healthy subjects (35F/29M) aged 25–50	NS	Supine Lateral Prone	Adjustable bed system with eight sectors that allowed the sector height to be controlled by subjects to achieve the most comfortable feeling without adjustment with adjustment	NA
Verhaert et al. (2011)	17 healthy subjects (8F/9M), age mean = 24.3 ± 7.1	Insomnia, medical problems that interfere with normal sleep, back pain	No control, biomechanical measurement on lateral posture only	Dynasleep, mattress equipped with indentation sensors and adaptive actuator spring pockets Actuator active, induced different stiffness in eight zones to optimize spinal curvature based on the results of indentation measurements Manually adjust the actuator to simulate a sagging support (high stiffness at shoulder zone, low stiffness at the waist and hip zones)	Custom8, Leuven, Belgium
Verhaert et al. (2012a)	65 subjects (33F/32M), age mean: 27.3 ± 11.5 Validation: subgroup of 20 subjects (8F/12M), age mean: 22.9 ± 3.8	NS	Supine Lateral Prone	Dynasleep, mattress equipped with indentation sensors and adaptive actuator spring pockets actuator active, induced different stiffness in eight zones according to anthropometric measurements and BMI manually adjust the actuator to simulate a sagging support	Custom8, Leuven, Belgium
Verhaert et al. (2012b)	18 subjects (9F/9M), age mean = 28.5 ± 4.7	NS	Lateral	Three types of bed base Homogeneous box-spring Multi-zone slatted base Multi-zone mesh base Three types of mattress Multi-zone pocket spring mattress Multi-zone latex mattress Homogeneous PU foam mattress (nine combinations)	NS
Verhaert et al. (2013)	18 subjects (8F/10M), age mean = 31.3 ± 14.3 Field study: 12 subjects (6F/6M), age mean = 38.7 ± 23.4	Medical problems that interfere with normal sleep, back pain, sleep medications, antidepressants	No control, postures were detected and estimated in system configuration; six sets of postures in a field study (supine, left/right lateral, prone, intermediate left/right)	Dynasleep mattress equipped with indentation sensors and adaptive actuator spring pockets	Custom8, Leuven, Belgium
Wu, Yuan & Li (2018)	17 healthy subjects (4F/13M), age mean: 34.9 ± 9.7	Backache in the last 10 days, any spinal deformations	Supine	Palm fiber Bilayer, upper layer: latex, lower layer: palm fiber Palm fiber, Young’s modulus *E* = 46.73 ± 5.72 kPa. Latex, hyperelastic Ogden’s parameter, μ = 1.28 ± 0.13 kPa, α = 4.175 ± 0.885, β = 0.314 ± 0.048	Guizhou Nature Technology Co., Ltd., Guiyang, China NS
Yoshida, Kamijo & Shimizu (2012)	14 male college students aged 21–24 Finite element model: three subjects were picked from the pool to form the best body dimension coverage	NS	Supine	Four types of pocket coil mattress with *E* = 14.0 kPa *E* = 11.4 kPa *E* = 9.6 kPa *E* = 6.0 kPa	NS
Zhong et al. (2014)	Nine females classified into three groups (*n* = 3) based on BMI	Diagnosed musculoskeletal pathology	Supine	A total of 14 mattresses formed by the different combination of regional stiffness in five zones using six types of spring stiffness. The mattress consisted of a superficial layer of PU foam and a core layer composed of rows of pocketed springs.	NA

**Notes:**

M, male; F, female; BMI, body-mass index; PU, polyurethane; NA, not applicable; NS, not specified.

* The authors used the unit of kPa to describe the density of the sleeping support without justification.

**Table 2:** Methods and outcomes of biomechanical measurements in the reviewed articles.

Author (Year)	Measurement	Instrument (methods*)*	Manufacturer	Biomechanical measurement outcome
Chen et al. (2014)	Body-mattress contact pressure Sleep quality/polysomnography Subjective feedback	ABW body pressure measurement system ALICELE PSG polysomnograph Questionnaire, yes/no questions on hardness, comfortability, and difficulty to fall asleep	NS Philips Co., Andover, USA NA	Max pressure, min pressure, total stressed area
Denninger, Martel & Rancourt (2011)	Body dimensions Body mass distribution Force-compression curve of foam cubes loaded with body volume slice Spinal curvature	POWERSHOT A610 camera Custom-made apparatus with load cells ANSYS, finite element method Optotrak 3020 optical measurement system	Canon, Ontario, Canada NS Ansys Inc., Pittsburgh, USA Northern Digital Inc., Ontario, Canada	Location of vertebra, mass of each body volume slice, force-compression curve of individual mattress cubes
Deun et al. (2012)	Body surface contour Sleep quality/polysomnography Spinal curvature Subjective feedback	IKÉLO optical measurement system Dream system, polysomnograph Indentation sensors embedded in Dynasleep mattress (Spinal curvature was simulated and estimated by indentation using a human model personalized based on the results of body contour measurements) Questionnaires:Karolinska sleepiness scale, profile of mood state, stress/arousal adjective checklist, activation/deactivation adjective checklist	Custom8, Leuven, Belgium Medatec, Brussels, Belgium Custom8, Leuven, Belgium NA	NA (The results of biomechanical measurement were not included)
Esquirol Caussa et al. (2017)	Body dimensions Body-mattress contact pressure	Kinect camera and tape Surface with integrated pressure capacitive sensors	Microsoft, Washington, USA NS	Number of pressure points exceeded the threshold level in head and body regions
Lee et al. (2015)	Body-mattress contact pressure Subjective feedback	Body pressure measurement system Questionnaires: pain score using visual analogue scale, faces pain rating scale, Iowa pain thermometer	Tech Storm, DaeJeon, Korea NA	Mean pressure in different body regions (head, shoulder, right/left arm, lower back, pelvic girdle, right/left leg)
Lee et al. (2016)	Body-mattress contact pressure Subjective feedback	Body pressure measurement system Questionnaires: pain score using visual analogue scale, faces pain rating scale, Iowa pain thermometer	Tech Storm, DaeJeon, Korea NA	Mean pressure in different body regions (head, shoulder, right/left arm, lower back, pelvic girdle, right/left leg)
Leilnahari et al. (2011)	Spinal curvature	(1a) DCR-TRV356E cameras (1b) BRG.LIFEMOD2007, musculoskeletal modeling*(spinal curvature was simulated and estimated by modeling and validated by captured images)*	(1a) Sony Co., Tokyo, Japan (1b) LifeModeler, San Clemente, US	Location of vertebra centreπ-P_8_: angle between the thoracic spinal line and the lumbar spinal line
López-Torres et al. (2008)	Mannequin-mattress contact pressure Subjective feedback	PLIANCE 19 P body pressure measurement system Questionnaire: perceived firmness with hands, buttocks, in supine/lateral posture; difficulties in rolling over and getting up; four-point grading in comparing overall comfort	Novel, Munich, Germany NA	Max pressure; average pressure; average contact area
Low et al. (2017)	Body-mattress contact pressure	TEKSCAN 5400N pressure mapping sensor	Tekscan, South Boston, USA	Peak body contact pressure and contact area in back torso and buttocks for supine; side torso (inclusive upper arm and shoulder) for lateral; front torso (chest and stomach) for prone
Palmero et al. (2017)	Body surface contour Body-mattress contact pressure	Kinect camera In-house built capacitive pressure-sensitive mattress sensor	Microsoft, Washington, USA NS	Number of pressure points exceeded the threshold level in head and body regions
Park, Kim & Kim (2009)	Body-mattress contact pressure Subjective feedback	Self-assembled force-sensing resistor matrix Questionnaire, five-point scale of comfortability in nine body regions (neck, shoulder, back, elbows, lumbar, hand/wrist, hip/thigh, knee, ankle)	NS NA	Fraction of body pressure on eight transverse bed sectors
Verhaert et al. (2011)	Body dimensions Body surface contour Spine curvature Sleep quality/polysomnography Subjective feedback	Calliper and tape IKÉLO optical measurement system 3D Vario rasterstereograph (*Spinal curvature was estimated using an algorithm based on body dimension and surface measurements)* Dream System, polysomnography Questionnaire: Karolinska sleepiness scale, arousal scale of Cox’s stress, arousal adjective checklist, profile of mood states.	NS Custom8, Leuven, Belgium Vialux, Chemnitz, Germany Medatec, Brussels, Belgium NA	P_1_: angle between the VP-DM line and the horizontal axis; P_2_: mean distance between the spinal curvature line and its least square line; P_3_: angle between the least square line and the horizontal axis; P_4_: angle between thoracic and lumbar least square lines.
Verhaert et al. (2012a)	Body dimensions Body surface contour Spinal curvature	Calliper and tape IKÉLO optical measurement system Indentation sensors embedded in Dynasleep mattress. *(Spinal curvature was simulated and estimated by indentation using a human model personalized based on the results of body contour measurements)*	NS Custom8, Leuven, Belgium Custom8, Leuven, Belgium	P_1_: angle between the pelvis-shoulder line and the horizontal line; P_2_: angle between the least square line of the spine curvature and the horizontal line; P_3_: angle between thoracic and lumbar least square lines.
Verhaert et al. (2012b)	Body surface contour Body surface contour *(for validation)* Spinal curvature	IKÉLO optical measurement system zSnapper 3D scanner Spinal curvature was simulated and estimated based on the mass distribution of body portions and the human model personalized by body surface measurements and validated by 3D scanning	Custom8, Leuven, Belgium Vialux, Chemnitz, Germany NA	Least square line of spinal points (α); angle between lumbar and thoracic parts of the spine (γ). The score (EBS_L) featured a weighted combination of α and γ
Verhaert et al. (2013)	Spinal curvature	Indentation sensors embedded in Dynasleep mattress. *(Spinal curvature was estimated using indentation data and a personalized human model)*	Custom8, Leuven, Belgium	P_1_: angle between the horizontal line and the line connecting starting and ending points of the spine; P_2_: mean unsigned distance from the spine to its least square line; P_3_: angle between the horizontal line and the least square line; P_4_: angle between the thoracic and lumbar least square line; P_5_: RMSD between the spine curvature and the reference spine; P_6_: difference between the lordotic angle of the spine curvature and the reference spine; P_7_: difference between the kyphotic angle of the spine curvature and the reference spine; P_1_–P_4_ for lateral posture; P_5_–P_7_ for supine posture.
Wu, Yuan & Li (2018)	Back surface contour Spinal alignment/mattress indentation Body-mattress contact pressure	3D body scanning system ANSYS finite element model Tactilus body pressure measurement system	NS; ANSYS Inc., Pennsylvania, US; Sensor Products Inc., Madison, US	Max pressure, total pressure and the contact area of thoracic, lumbar and buttock regions;*L*_LT_, *L*_LB_: thoracic-lumbar and lumbar-buttock distances between standing and supine lying; similarity of back surface contour between measured upright standing and predicted supine lying.
Yoshida, Kamijo & Shimizu (2012)	Internal stress, head & chest displacement Subjective feedback	ANSYS finite element model Questionnaire, seven-grade scale on the feeling of firmness, mattress preference, firmness preference, sinking preference, comfort for different regions of the body	ANSYS Inc., Pittsburgh, USA; NA	Von Mises stress of cervical vertebra; sinking displacement of the head and chest
Zhong et al. (2014)	Spinal curvature	Custom-made indentation measuring bar embedded in the mattress *(Spinal curvature was estimated by fitting a curve on the indentation points)*	NA	Back-inclination line: line joining the lower points of the curve at the upper back and the hip; back-hip inclination angle (β): angle between the back-inclination line and the horizontal axis; CTh, ThL, LS (angle between the region line and the back-inclination line); depth of lumbar lordosis (DL)

**Note:**

NA, not applicable; NS, not specified; VP, vertebral prominens; DM, the midpoint of the dimples of the posterior superior iliac spine; RMSD, root mean square distance; CTh, cervicothoracic angle; ThL, thoracolumbar angle; LS, lumbosacral angle.

**Table 3:** Study design and scope of the reviewed articles.

Author (Year)	Study design	Scope/objective	Key findings
Chen et al. (2014)	Randomised cross-over, single-blind controlled trial	To investigate the influence of mattress stiffness on body contact pressure and sleep quality.	Polysomnographic analysis and subjective feedback revealed that a mattress with an intermediate level of contact pressure exhibited better sleep quality.
Denninger, Martel & Rancourt (2011)	Design process, validation of simulation (deviation)	Design of a customized mattress based on optimized spinal curvature in the frontal plane and minimization of trunk shear force; Development and validation of a simplified finite element model for the design process.	A design process comprising a look-up table of human-mattress interaction predicted by simulation was established. The design of a customized mattress with different cube cavity dimensions could be defined together with the input of body properties.Validation showed a load distribution within a 10% average deviation from the expected distribution; spine alignment was within a distance of ±3% shoulder width from the expected spine curvature.
Deun et al. (2012)	Repeated measures, non-randomized controlled trial	Investigation of sleep quality induced by an active-control bedding system that autonomously alters stiffness distribution according to the estimated spinal alignment, as compared to the inactive mode of this system	When active control mode was used, sleep quality was significantly improved, as revealed by polysomnographic analysis and subjective feedback.
Esquirol Caussa et al. (2017)	Recommendation model, validation of somatotype model (correlation)	Design and validation of an automatic multimodal somatotype determination model to automatically recommend mattress-topper-pillow design combinations.	Validation of somatotype models demonstrated a high correlation index compared to real data: more than 85% in height and body circumferences; 89.9% in weight; 80.4% in body mass index; and more than 70% in morphotype categorization.
Lee et al. (2015)	Mixed factorial design (gender, body regions, duration), non-randomized controlled trial	Analysis of body pressure and perceived level of pain for different genders, body regions, and durations of supine lying.	Head regions experienced significantly higher pain scores and pressure intensities; lower back was not too high in pressure intensity but featured the second highest pain score; the back and pelvic girdle showed a significant difference between males and females on the pain score; pain appeared in all body regions after 10 min and progressed as time increased.
Lee et al. (2016)	Repeated measurements, non-randomized controlled trial	Comparison of body pressure and perceived level of pain between the floor and mattress for different durations of supine lying.	Head regions featured a significantly higher pressure intensity; the pain scores of all body regions except for legs were significantly higher for the floor condition; the pain score of the floor condition significantly increased at 1 min compared to those of the mattress group.
Leilnahari et al. (2011)	Design process, repeated measurements, non-randomized controlled trial	Design of a customized mattress with different zonal elasticity that can achieve optimal spinal alignment; Comparison of spinal alignment achieved by firm, soft, and custom mattresses.	The customised mattress with different zonal elasticity afforded better spinal alignment (least π-P_8_), followed by firm and soft mattresses.
López-Torres et al. (2008)	Non-randomized controlled trial, correlation	Comparison of perceived firmness, usability, and comfort between young and elderly people; Investigation of the correlation between subjective ratings and results of objective measurements (pressure distribution and objective firmness).	No perception differences between the young and the elderly were found. Significant correlations were found between increments in objective firmness and perceived firmness (positive); increments in average pressure and perceived firmness (positive); increments in objective firmness and average pressure were associated with increments in overall comfort and reductions in rolling difficulty.
Low et al. (2017)	Randomized cross-over, single-blind controlled trial	Comparison of the body contact pressure profile of different mattresses in three different postures.	Compared to the case of a PU mattress, reduced peak pressure and a more even pressure distribution was observed for a latex mattress.
Palmero et al. (2017)	Recommendation model, validation for morphotype categorization (confusion matrix, correlation)	Development and validation of a somatotype determination model based on 3D RGB-depth imaging (Kinect) and automatic landmark points extraction; Establishment of a recommendation model for mattress-topper-pillow design combinations based on somatotype model and pressure analysis.	The system was capable of accurate categorization and achieved high correlation results with respect to manual measurement.
Park, Kim & Kim (2009)	Design process, repeated measurements, non-randomized controlled trial	Development of an adjustable bed that regulates the height of eight mattress sectors and allows self-adjustment;Comparison of adjustable bed and flatbed comfort ratings.	Subjects preferred height adjustment in W-shape in supine and lateral postures, and in U-shape in lateral prone postures; The adjusted height was significantly correlated with (a) the subjective rating and (b) the ratio of bed sector regional pressure and the total bed pressure.
Verhaert et al. (2011)	Repeated measurements, non-randomized controlled trial	Investigation of the effect of an active-control bedding system autonomously altering stiffness distribution according to the estimated spinal alignment and comparison to a sagging bedding system.	The sagging sleep system negatively affected sleep quality in prone and lateral postures; The relationship between mattress design and sleep quality was affected by anthropometry and posture.
Verhaert et al. (2012a)	Instrument design, validation (correlation)	Development of an estimation method for spinal alignment by integration of a personalized human model and mattress indentation measurements.	Good intraclass correlation (0.73–0.88) between estimated and measured angular spinal deformation was observed.
Verhaert et al. (2012b)	Instrument design, validation (deviation), recommendation model	Estimation of spinal shape using a personalized anthropometric model and load-deflection characteristics of the mattress and bed base;Presentation of a method to identify mattress bed base combinations with superior support properties.	Estimation showed good correspondence (85%) in comparison to the validated spine shape in terms of score ranking.
Verhaert et al. (2013)	Mattress design process, randomized crossover single-blind controlled trial	Presentation of an active-control mattress system that can: detect body movement and recognize sleep posture; estimate the shape of the spine by combining indentation with human models; based on indentation measurement and feedback, control the mattress system to achieve optimal spinal alignment by customizing regional mattress stiffness. Performance comparison of the active and non-active modes of the active-control mattress.	The use of the active-control mattress system significantly improved the perceived sleep quality.
Wu, Yuan & Li (2018)	Instrument design, repeated measurements	Development of a mattress evaluation method based on body pressure distribution and comparison of back surface and spinal alignment between supine lying and upright standing through finite element simulation.Comparison of the outcomes obtained for a palm fiber mattress and a bilayer latex/palm fiber mattress.	A novel parameter was proposed by comparing the back surface contours of supine lying and natural standing postures via similarity analysis. The bilayer latex/palm fiber mattress produced a back surface contour close to that of upright standing, which indicated a preferable selection.
Yoshida, Kamijo & Shimizu (2012)	Correlation (simulation vs. subjective rating)	Investigation of the relationship between the outcome of computer simulation (finite element analysis) and subjective ratings on preference and comfort.	The subjective ratings corresponded to the prediction outcome, including the von Mises stress of the cervical vertebral region and the sinking displacement of the neck region.
Zhong et al. (2014)	Instrument design, validation (error analysis), mattress design process	Estimation of spinal curvature with mattress indentation; Determination of an optimal mattress zonal stiffness.	The overall mean absolute error and mean relative error between the estimation and experimental measurements equaled 3.4 mm (SD: 2.7) and 9.27%, respectively. CTh, ThL, LS generally increased with lower back and hip zone stiffening; the upper body became more levelled with stiffened hip zones and more inclined with stiffened upper back zones.

**Note:**

PU, polyurethane; CTh, cervicothoracic angle; ThL, thoracolumbar angle; LS, lumbosacral angle; SD, standard deviation.

## Results

### Subjects and population

In the reviewed articles, all participants were normal subjects, since the review was confined to the use of domestic mattresses. Subjects with sleep disorders, related medications, or musculoskeletal disorders, for example, spine and back pain, were excluded. However, no study addressed the assessment methods of these criteria. While we assumed that the exclusion process was based on self-reports, both sleep disorders and musculoskeletal problems could be often undiagnosed (Behar et al., 2013; Woolf, Erwin & March, 2012).

Two studies investigated only male subjects, and one study investigated only female subjects. No study attempted to compare the differences between males and females, despite the fact that gender can influence body built, joint stiffness, and comfort perception.

Although young adults were commonly recruited, López-Torres et al. (2008) compared the outcomes obtained for young adults and elderly people, revealing the absence of significant differences between these groups in terms of subjective perception. The works of Esquirol Caussa et al. (2017) and Palmero et al. (2017) encompassed a wide age range (4–93) of subjects, but the influence of age was not discussed in either case.

Considering the influence of body built, Esquirol Caussa et al. (2017) identified five body morphotypes based on body girth to determine individualized sleeping system recommendations. Additionally, body dimensions were used to estimate the customized configuration of mattress zonal stiffness, although its influence on the biomechanics outcome was not documented (Verhaert et al., 2011b, 2012a). Finally, several studies confirmed that the required mattress stiffness depended on body form and spinal curvature (Yoshida, Kamijo & Shimizu, 2012; Zhong et al., 2014).

### Mattress samples

Among the 18 articles, eight focused on market-available ordinary mattresses (Table 1), for example, Esquirol Caussa et al. (2017) explored mattress-topper-pillow combinations with different material densities (although the use of pressure units (kPa) for density quantification was not explained), while Yoshida, Kamijo & Shimizu (2012) reported the Young’s moduli of four types of pocket coil mattresses. In some studies, mattress samples were only vaguely described. López-Torres et al. (2008) selected four mattresses claimed to cover the full range of firmness, while Low et al. (2017) commented that the density of their mattresses was intermediate. Palmero et al. (2017) used the subjects’ existing mattresses but did not describe their brand, stiffness, or type.

Five articles involved the development of specialized or customized mattresses. Denninger, Martel & Rancourt (2011) packed cells of cubes with hollow ellipsoidal cavities and customized the dimensions and cavities of these cubes based on individuals’ spinal curvature and regional body weight portion. Similarly, Chen et al. (2014) fitted the mattress intermediate layer with the body shape contour, and Park, Kim & Kim (2009) allowed the subjects to adjust the mattress regional height according to their own preferences. Some studies customized mattress regional stiffness to achieve desirable spinal curvature. For example, Leilnahari et al. (2011) and Verhaert et al. (2012a). In addition, Zhong et al. (2014) divided the mattress into five zones and evaluated 14 combinations using six types of spring stiffness in different zones.

Five of the selected articles employed the same active-control mattress system, Dynasleep (Custom8, Leuven, Belgium) (Deun et al., 2012; Verhaert et al., 2011b, 2012a, 2012b, 2013), which was equipped with indentation sensors and adaptive actuator spring pockets, with each mattress cell containing two pocket springs with different intrinsic stiffness coefficients (*k* = 0.2 and 0.076 N/mm) arranged in a parallel way. Real-time adjustment of regional stiffness was facilitated by the vertical displacement of spring pockets according to sensor-supplied data and the pre-set algorithm. The articles did not disclose the detailed algorithm of the active control design, although the relevant content could be found in the dissertation of one of the authors (Verhaert, 2011).

Esquirol Caussa et al. (2017) and Palmero et al. (2017) developed a recommendation matrix for suggesting mattress-pillow-topper combinations. Although the interactions of these components were not studied, the inclusion of different support components is appreciated, since other studies seldom specify the use of pillows and toppers. Importantly, pillow design has a compelling effect on the biomechanics of sleep support, particularly on the cervical spine posture (Ren et al., 2016).

### Posture

Supine and side lying postures are frequently evaluated in literature. Most of such studies are controlled trials in which the posture is instructed and maintained during the required time period, for example, for supine posture, subjects were asked to put their hands straight on both sides. In the work of Esquirol Caussa et al. (2017), subjects were instructed to put their feet together, while in the work of Palmero et al. (2017), legs were placed slightly apart. For lateral posture, subjects were normally placed with the body perpendicular to the ground surface (Denninger, Martel & Rancourt, 2011; Leilnahari et al., 2011), which was challenging, since people tend to turn their shoulder forward toward the mattress (Verhaert et al., 2013). Additionally, limb placement was subject to some variations. In some studies, limb flexion was controlled at a given angle (Leilnahari et al., 2011), while in others, subjects were allowed to bend the limb slightly and naturally (Denninger, Martel & Rancourt, 2011). The variation of sleep posture may produce different trunk bending angles and thus may influence the outcome of the spinal alignment. The influence of the posture variation requires further investigation. Prone posture was relatively less evaluated and specified (Low et al., 2017).

Instead of adopting an experimental approach, some studies simulated and estimated postures using computational models. By scaling a simplified model with subject body dimensions, Verhaert et al. (2012a, 2012b) attempted to evaluate supine, lateral, and prone lying postures on ordinary, customized, and active-control mattresses. Zhong et al. (2014) conducted a finite element analysis using a supine lying human model to assess the influence of mattress regional stiffness variability.

The use of posture detection and prediction algorithms allowed the conduction of observational studies with uncontrolled sleeping postures. Verhaert et al. (2011b) used cluster analysis to categorize the population into two groups according to the time spent in lateral, prone, and supine postures, while Deun et al. (2012) and Verhaert et al. (2013) identified four main sleeping postures (including left- and right-side lying, prone lying, and supine lying) employing indentation data provided by the active-control bed. The above categorization was achieved using a support vector machine according to the combination of indentation, shoulder-hip ratio, knee-hip ratio, total indentation, lateral asymmetry index, and lower leg indentation data, which has been proven to achieve sleeping posture recognition with >90% accuracy (Verhaert et al., 2011a). However, this technique was not sufficiently sensitive to detect intermediate postures, which account for 10% of the sleeping time (Verhaert et al., 2013).

### Determinants and evaluation methods

Although body-mattress contact pressure is commonly used to represent the entity of pain and discomfort, the validity of this approach remains controversial (Buckle & Fernandes, 1998). Force or pressure induces the deformation of skin and thus triggers the sensation of touch (via mechanoreceptors) and pain (via nociceptors) upon high loading (Kilinc-Balci, 2011). High pressure can also adversely affect peripheral blood circulation and lead to numbness and discomfort (López-Torres et al., 2008). Therefore, the perception of pain or comfort is believed to be strongly related to the perception of pressure (López-Torres et al., 2008). The body pressure measurement system was characterized by thin and flexible sheet sensors that only minimally interfered with the mattress support (Chen et al., 2014; Lee et al., 2015, 2016; Low et al., 2017). Yet, the sensors may disperse the concentrated pressure and thus underestimate the peak pressure. Similarly to the principle of contact pressure, some studies investigated the effects of the regional supporting load using a matrix of load cells (Denninger, Martel & Rancourt, 2011) or an indentation bar (Zhong et al., 2014) and indentation sensors embedded in the mattress (Dynasleep). Peak pressure, average pressure, and contact area were often measured in different body regions with the aim of pressure reduction.

Spinal alignment was the second frequently investigated parameter, since the adoption of neutral or physiological spine curvature is thought to avoid musculoskeletal problems or pain. In fact, spinal alignment or curvature in side-lying postures was frequently evaluated because of the ease of measurement. Several studies limited the measurement of alignment to two dimensions at the coronal plane using a camera (Denninger, Martel & Rancourt, 2011; Leilnahari et al., 2011), while some attempted to perform three-dimensional (3D) measurements using pen-tip optical tracking (Denninger, Martel & Rancourt, 2011), rasterstereography (Verhaert et al., 2011b), a camera equipped with a depth sensor/infrared projector (Kinect, Microsoft, Redmond, WA, USA) (Esquirol Caussa et al., 2017; Palmero et al., 2017), and registration of images from sagittal and coronal planes (Deun et al., 2012; Verhaert et al., 2011b, 2012a, 2012b).

Zhong et al. (2014) approximated spinal curvature in the supine position using a custom-made indentation bar embedded in the mattress, while others attempted to model the supine spine by integration of indentation measurements and computer modeling. Human models were personalized by scaling a generic model with measured body dimensions or 3D scanning (Deun et al., 2012; Leilnahari et al., 2011; Verhaert et al., 2011b, 2012a, 2012b, 2013; Wu, Yuan & Li, 2018). Instead of using a simple soft-tissue-lump model, Verhaert et al. (2013) combined the body surface model with a simplified skeleton model to enhance the accuracy of posture approximation, and further improvement was achieved by Leilnahari et al. (2011) through the use of a musculoskeletal model (BRG.LifeMod) accounting for joint stiffness and the range of motion.

There was no consensus on the use of a specific parameter to quantify spinal alignment. For example, a simple approach was used in two cases to identify the locations of each vertebra center (Denninger, Martel & Rancourt, 2011; Leilnahari et al., 2011), while in other cases, the thoracic-lumbar angle was estimated using the regression lines of thoracic and lumbar regions (Leilnahari et al., 2011; Verhaert et al., 2011b, 2012a, 2012b), since a discontinuity was often observed at the transition from the flexible lumbar to the rigid thoracic regions (Leilnahari et al., 2011). Some angles were also calculated by comparing lines joining the upper and lower regions with the horizontal line (Verhaert et al., 2011b, 2012b, 2013; Zhong et al., 2014). Mean distances were measured between the regressed curved line and the horizontal line, the line of the spinal axis, or a reference curve (Verhaert et al., 2011b, 2013; Zhong et al., 2014), and root-mean-square deviations were computed to quantify deflections from the desired curvature. However, no study investigated the twisting of the spine or trunk segments, which can be overlooked in some occasions of back pain.

Finite element analysis provides a versatile platform to predict the internal biomechanics of the body in a controlled environment (Wong et al., 2014). Regarding internal stress and strain, Yoshida, Kamijo & Shimizu (2012) performed finite element analysis to examine the von Mises stress of the second to fifth cervical vertebra for different mattress firmness, additionally comparing sinking displacements in head and thoracic regions. Wu, Yuan & Li (2018) modeled the back contour of the human body by finite element simulation and compared it with that observed during natural standing. In addition, Denninger, Martel & Rancourt (2011) constructed a simplified finite element model of the whole body and optimized the design of mattress cells by equalizing the body portion weight with the supporting force of each mattress cell. The process was performed assuming a minimal trunk shear force predicted by finite element analysis. The limitations of finite element simulation include model simplifications and assumptions on pre-defined sets of loading cases (Wong et al., 2017). It remains difficult to reconstruct a few anatomically detailed models with corresponding experiments for validation (Wong et al., 2018). Oversimplified models could have problems on the prediction accuracy and validity that could limit practical applications (Wang, Wong & Zhang, 2016). Non-biomechanical methods were implemented to correlate biomechanical parameters with sleeping quality and subjective feedback on comfort using polysomnography and questionnaires. The details of these methods are beyond the scope of this review and can be found elsewhere (Radwan et al., 2015).

### Optimization or selection criteria

Since contact pressure and spinal curvature/alignment are the predominant parameters of interest, it is important to know the desirable range or values of these parameters to suggest the directions of design optimization and realize high-quality mattresses.

Mattress design often strives for lower contact pressure in view of the fact that high pressure may cause discomfort and sore formation (Esquirol Caussa et al., 2017; López-Torres et al., 2008; Low et al., 2017; Palmero et al., 2017). Low et al. (2017) aimed to reduce peak pressure and realize a more even pressure distribution. Additionally, Esquirol Caussa et al. (2017) and Palmero et al. (2017) concluded that a soft topper should be implemented when more than three points with pressure exceeding 60 mmHg are present, while a medium-density pillow should be used when the maximum pressure at the occipital region falls between 30 and 40 mmHg. Conversely, several authors opposed this view by showing that high pressure and discomfort should not necessarily be correlated (Lahm & Iaizzo, 2002; Lee et al., 2016). Chen et al. (2014) commented that to achieve better sleep quality, the body pressure distribution should neither be over-concentrated nor over-distributed. Different body regions can exhibit different pressure tolerabilities (Lee et al., 2015), while the comfort of mattresses with different stiffness can be perceived differently depending on body built or body weight (Yoshida, Kamijo & Shimizu, 2012).

A straight horizontal line in the frontal plane was employed for evaluating spinal alignment in a lateral lying posture. A scoliotic spine position was regarded as non-natural or non-physiological and was believed to result in muscle imbalance and back pain (Aebi, 2005). The S-shaped curvature of the spine in the sagittal plane was of particular interest for supine lying. Denninger, Martel & Rancourt (2011), Wu, Yuan & Li (2018), and Zhong et al. (2014) assumed upright spine alignment or curvature as the desired alignment, while Verhaert et al. (2013) stated that the targeted upright spine should consider a slightly flattened lumbar lordosis to accommodate the switched working axis of gravity. In addition to spine alignment, Wu, Yuan & Li (2018) compared the back surface contour obtained for simulated supine lying with that determined by 3D scanning during natural standing and proposed a comfort index based on similarity analysis.

Compromising and weighing of two or more determinants to establish a single measure remains difficult. Verhaert et al. (2012a) formulated an ergonomic bed score (EBS_L) based on a weighted combination of lumbar and thoracic angles, whilst Denninger, Martel & Rancourt (2011) presented an expert system that considered both spinal curvature and trunk shear during the design process. However, neither of these authors considered the trade-off between such criteria and objective functions. The study of Wu, Yuan & Li (2018) was the only one that considered multiple dimensions such as body pressure, back surface contour, and spine alignment. The obtained results showed that these parameters provided conflicting conclusions toward better mattress construction, and it was decided to use the back surface contour as the determinant after alleged comprehensive consideration.

### Study design and key findings

The reviewed articles had diverse study objectives and hence, study designs, as shown in Table 3. Five articles investigated the processes and methods of designing new mattresses, including those with customized regional stiffness and height (Denninger, Martel & Rancourt, 2011; Leilnahari et al., 2011; Park, Kim & Kim, 2009; Verhaert et al., 2013; Zhong et al., 2014). Custom-made mattress that was constructed with different zonal elasticities produced the smallest thoracolumbar angle (4.10°) compared to the firm (8.9°) and soft surface (12.66°) (Leilnahari et al., 2011). Zhong et al. (2014) suggested that a custom-made mattress with stiffening of the lower back and hip regions would increase the cervicothoracic, thoracolumbar, and lumbosacral angles, while the stiffening of the upper back region would decrease these angles. Park, Kim & Kim (2009) allowed the subjects to adjust the regional heights of the mattress and discovered that there was a significant correlation between the regional pressure ratio differences and subjective ratings. They preferred the W-shaped bed in both supine and side postures and U-shaped bed in prone posture, compared to the flat bed (Park, Kim & Kim, 2009).

Using different objective functions, three research teams developed recommendation models for the optimal selection of mattresses or combinations of sleep system constituents (pillow, mattress, and topper) (Esquirol Caussa et al., 2017; Palmero et al., 2017; Verhaert et al., 2012a). Another three studies involved the design of instruments or techniques to estimate spinal curvature during supine lying, which is otherwise difficult to assess because of the lack of back exposure (Verhaert et al., 2012a, 2012b; Zhong et al., 2014).

The introduction of new instruments and the application of computer simulations call for a validation process. Percentage errors or deviation values were common and simple parameters used for validation (Denninger, Martel & Rancourt, 2011; Verhaert et al., 2012a; Zhong et al., 2014), while some studies conducted correlation analysis and introduced confusion matrices (Esquirol Caussa et al., 2017; Palmero et al., 2017; Verhaert et al., 2012b).

Comparative studies were conducted to evaluate conventional and newly designed mattresses. Chen et al. (2014) and Low et al. (2017) implemented a randomized cross-over single-blind controlled trial to evaluate different mattress materials. Low et al. (2017) found that latex mattresses can significantly reduce peak pressure by up to 35.1% compared to that of the polyurethane mattresses. Besides, mattresses with over-concentrated or over-even pressure distribution produced low satisfaction scores and were proven not beneficial to sleep quality (Chen et al., 2014).

Deun et al. (2012) and Verhaert et al. (2011b, 2013) evaluated an active-control bed by comparing it to that with a non-active mode or an exaggerated sagging condition by repeated measurements. Their results demonstrated that the active-control bed significantly improved subjective ratings (sleep quality, daytime quality, perceived number of awakenings), polysomnographic measurements and spinal alignment.

### Remarks

Herein, we reviewed the state-of-the-art biomechanical research on sleeping mattress design, particularly its scope and methodology, demonstrating that mattress research and development have shifted from homogeneous material evaluation to regional characteristic customization. Several authors attempted to adjust the height or stiffness of different zones to facilitate the adoption of physiological spine curvature. An active-control bed system was shown to enable regional stiffness change in real time to accommodate different postures, and several studies used the same active-control bed system but employed slightly different control algorithms for a better response. In fact, a market research conducted by TechNavio identified smart mattresses with sleep tracking, movement detection, and automatic firmness adjustment functions as the next major market driver in the mattress production industry.

To date, pressure is one of the gold standards for mattress performance evaluation, despite providing results that are in subtle conflict with those obtained using sufficient supporting force as an evaluation parameter. For instance, low pressure can be used to improve tactile comfort at the cost of a sagging spine and sinking lumbar region, while the introduction of expensive support surfaces to eliminate pressure is not necessarily beneficial (Goossens, 2009; Lahm & Iaizzo, 2002). Esquirol Caussa et al. (2017) and Palmero et al. (2017) aimed at a pressure threshold of 30–40 mmHg, with attenuation performed by varying the density of pillows and toppers. The threshold value was defined according to the premise that subcutaneous ischemia happens at a critical capillary close pressure of >30 mmHg (McCall, Boggs & Letton, 2012). However, it should be noted that this assumption of equating capillary pressure and skin contact pressure may not be valid for the trunk, which features thick soft tissues and muscles. In fact, in relevant studies, measurement were performed at the fingernail fold (De Graaff et al., 2002). Lee et al. (2015, 2016) demonstrated that different body regions feature different pressure sensitivity and tolerability, highlighting the need to establish a reference quantitative range of acceptable pressure at different body regions for mattress design.

Spinal alignment is believed to reflect the complex biomechanical interaction between the human body and the sleeping support (Verhaert et al., 2012a). However, the measurement of spinal alignment in the supine position remains challenging because of the lack of back exposure and the fact that instrument placement may interfere with body support. Although such estimations are commonly performed by computer models referenced to the results of 3D scanning during upright standing, we proposed the use of optical Fiber Bragg Grating (FBG) sensors for an unobtrusive assessment of spine alignment (Sadek et al., 2017). In particular, the suggested flexible thin wire FBG sensor allowed real-time 3D geometry sensing without causing much interference to the support surface (Allsop et al., 2012; Ryu & Dupont, 2014) and thus enabled the detection of different planes including body twisting, which was often overlooked. Moreover, the FBG system can also sense temperature and humidity simultaneously, which are important attributes affecting sleeping comfort (Zhang et al., 2010).

Similarly, the objective functions or criteria of spine alignment in supine postures are questionable, although Verhaert et al. (2012a) viewed it as the primary metric of the overall body deformation. Moreover, whereas upright standing can be the most available posture to be referenced, it is theoretically inappropriate to regard upright standing as a desirable alignment for supine sleeping, since the spine loading modes during standing and lying are totally different. Under the influence of gravity, the curvature of the standing spine tends to be vertically exaggerated because of the induced compression, while that of the lying spine tends to be flattened (Verhaert et al., 2012a). The role of intervertebral discs, muscles, and ligaments is expected to be more prominent during standing, which affects spine curvature and the perception of relaxation and comfort. To this end, we have discovered that the spine alignment desirable for supine sleeping can be determined under free-floating conditions. During flotation therapy (flotation spa), participants lie in a tank filled with salt-saturated water in a sound-, light-, and temperature-controlled environment (Van Dierendonck & Te Nijenhuis, 2005). The high density of the saline is believed to provide a sufficient and appropriate buoyancy force to support the body and has been proven to bring about relaxation responses, including the sense of zero-gravity and reduced muscle tension (Van Dierendonck & Te Nijenhuis, 2005). We believe that this approach could be a possible alternative to establishing a desirable supine lying posture, whilst the water tank can also allow exposure for measurement.

Despite the fact that most studies attempted to ease discomfort only, comfort is not exactly the opposite of discomfort (Goossens, 2009). Helander (2003) defined comfort as a sense of relaxation and relief. Likewise, Verhaert et al. (2012b) argued that comfortable bedding should facilitate the relaxation of muscles and intervertebral discs to recover from day-long loading. The lack of skeletal support was also regarded as a consequence of prolonged muscle activation that triggered a guarding action from the brain (Russell, 1999). However, it remains difficult to measure muscle loading and the deformation of intervertebral discs or other soft tissues during sleep. While computer simulations used in the reviewed articles employed simplified models to estimate spinal alignment, anatomically detailed finite element models and musculoskeletal models accounting for the functions of muscles and the geometry of the intervertebral discs can provide more complementary evidence on the objective representation of mattress comfort.

Mattress design and selection to achieve client satisfaction are recognized as a tedious trial-and-error process. This broad review was conducted to systematically sample the relevant information and thus improve the process of mattress design and selection as well as stimulate pertinent standard setup. While the studies implemented personalized design, cutting edge technology and algorithm to improve mattress design, the basic requirement of mattress design is inconclusive. It is pragmatically demanding to evaluate the influence of mattress material (stiffness), thickness, shape, and pillow combinations on the pressure distribution and spinal alignment systematically. This study features several limitations. First, we did not assess methodological quality, since the involved designs and scopes were diverse and thus difficult to compare. Second, a number of works dealt with anti-pressure-sore mattresses and were thus beyond the scope of this review. Moreover, eligible articles were determined by several manual screening processes and discussion, and the repeatability of the search and screening may thus be challenged.

## Conclusions

Future studies should aim to establish and justify reference values for mattress design and selection as well as develop an algorithm for determining the trade-off between the weighting of different determinants.

## Additional Information and Declarations

### Data Availability

The following information was supplied regarding data availability: This article is a literature review article and did not generate any data or code.

## References

[ref-1] Aebi M (2005). The adult scoliosis. European Spine Journal.

[ref-2] Allsop TD, Bhamber R, Lloyd GD, Miller MR, Dixon A, Webb DJ, Ania-Castañon JD, Bennion I (2012). Respiratory function monitoring using a real-time three-dimensional fiber-optic shaping sensing scheme based upon fiber Bragg gratings. Journal of Biomedical Optics.

[ref-3] Behar J, Roebuck A, Domingos JS, Gederi E, Clifford GD (2013). A review of current sleep screening applications for smartphones. Physiological Measurement.

[ref-4] Berlowitz DR, Brienza DM (2007). Are all pressure ulcers the result of deep tissue injury? A review of the literature. Ostomy Wound Management.

[ref-5] Buckle P, Fernandes A (1998). Mattress evaluation—assessment of contact pressure, comfort and discomfort. Applied Ergonomics.

[ref-6] Chen Z, Li Y, Liu R, Gao D, Chen Q, Hu Z, Guo J (2014). Effects of interface pressure distribution on human sleep quality. PLOS ONE.

[ref-7] Cimmino MA, Ferrone C, Cutolo M (2011). Epidemiology of chronic musculoskeletal pain. Best Practice & Research Clinical Rheumatology.

[ref-8] De Graaff JC, Ubbink DT, Lagarde SM, Jacobs MJHM (2002). The feasibility and reliability of capillary blood pressure measurements in the fingernail fold. Microvascular Research.

[ref-9] Denninger M, Martel F, Rancourt D (2011). A single step process to design a custom mattress that relieves trunk shear forces. International Journal of Mechanics and Materials in Design.

[ref-10] Deun DV, Verhaert V, Willemen T, Wuyts J, Verbraecken J, Exadaktylos V, Haex B, Vander Sloten J (2012). Biomechanics-based active control of bedding support properties and its influence on sleep. Work.

[ref-11] DeVocht JW, Wilder DG, Bandstra ER, Spratt KF (2006). Biomechanical evaluation of four different mattresses. Applied Ergonomics.

[ref-12] Esquirol Caussa J, Palmero Cantariño C, Bayo Tallón V, Cos Morera MÀ, Escalera S, Sánchez D, Sánchez Padilla M, Serrano Domínguez N, Relats Vilageliu M (2017). Automatic RBG-depth-pressure anthropometric analysis and individualised sleep solution prescription. Journal of Medical Engineering & Technology.

[ref-13] European Committee for Standardization (2000). EN 1957 standard: domestic furniture, beds and mattresses. Test methods for the determination of functional characteristics.

[ref-14] Goossens RHM, Gefen A (2009). Fundamentals of pressure, shear and friction and their effects on the human body at supported postures. Bioengineering Research of Chronic Wounds.

[ref-15] Helander MG (2003). Forget about ergonomics in chair design?. Focus on aesthetics and comfort! Ergonomics.

[ref-16] Kilinc-Balci F (2011). How consumers perceive comfort in apparel. Improving Comfort in Clothing.

[ref-17] Lahm R, Iaizzo PA (2002). Physiologic responses during rest on a sleep system at varied degrees of firmness in a normal population. Ergonomics.

[ref-18] Lee W-D, Lee J-U, Kim M-Y, Lee L-K, Park B-S, Yang S-M, Noh J-W, Shin Y-S, Kim J-H, Kwak T-Y, Lee T-H, Park J, Kim J (2016). Differences in the body pressure-related sensory changes between the floor and mattress in a static supine position for physiotherapy research: a randomized controlled pilot trial. Journal of Physical Therapy Science.

[ref-19] Lee W-D, Lee J-U, Park J, Kim J (2015). Analysis of the body pressure-related sensory changes in the static supine position for healthy science research: a randomized controlled pilot trial. Toxicology and Environmental Health Sciences.

[ref-20] Leilnahari K, Fatouraee N, Khodalotfi M, Sadeghein MA, Kashani YA (2011). Spine alignment in men during lateral sleep position: experimental study and modeling. Biomedical Engineering Online.

[ref-21] Liu S-F, Lee Y-L, Liang J-C (2011). Shape design of an optimal comfortable pillow based on the analytic hierarchy process method. Journal of Chiropractic Medicine.

[ref-22] López-Torres M, Porcar R, Solaz J, Romero T (2008). Objective firmness, average pressure and subjective perception in mattresses for the elderly. Applied Ergonomics.

[ref-23] Low F-Z, Chua MC-H, Lim P-Y, Yeow C-H (2017). Effects of Mattress material on body pressure profiles in different sleeping postures. Journal of Chiropractic Medicine.

[ref-24] McCall WV, Boggs N, Letton A (2012). Changes in sleep and wake in response to different sleeping surfaces: a pilot study. Applied Ergonomics.

[ref-25] Mork PJ, Vik KL, Moe B, Lier R, Bardal EM, Nilsen TIL (2013). Sleep problems, exercise and obesity and risk of chronic musculoskeletal pain: the Norwegian HUNT study. European Journal of Public Health.

[ref-26] Palmero C, Esquirol J, Bayo V, Cos MÀ, Ahmadmonfared P, Salabert J, Sánchez D, Escalera S (2017). Automatic sleep system recommendation by multi-modal RBG-depth-pressure anthropometric analysis. International Journal of Computer Vision.

[ref-27] Park SJ, Kim JS, Kim CB (2009). Comfort evaluation and bed adjustment according to sleeping positions. Human Factors and Ergonomics in Manufacturing & Service Industries.

[ref-28] Pearson EJM (2009). Comfort and its measurement–a literature review. Disability and Rehabilitation: Assistive Technology.

[ref-29] Radwan A, Fess P, James D, Murphy J, Myers J, Rooney M, Taylor J, Torii A (2015). Effect of different mattress designs on promoting sleep quality, pain reduction, and spinal alignment in adults with or without back pain; systematic review of controlled trials. Sleep Health: Journal of the National Sleep Foundation.

[ref-30] Ren S, Wong DW-C, Yang H, Zhou Y, Lin J, Zhang M (2016). Effect of pillow height on the biomechanics of the head-neck complex: investigation of the cranio-cervical pressure and cervical spine alignment. PeerJ.

[ref-31] Russell L (1999). A review of the medical agency services confor-med range. British Journal of Nursing.

[ref-32] Ryu SC, Dupont PE (2014). FBG-based shape sensing tubes for continuum robots.

[ref-33] Sadek I, Bellmunt J, Kodyš M, Abdulrazak B, Mokhtari M (2017). Novel unobtrusive approach for sleep monitoring using fiber optics in an ambient assisted living platform.

[ref-34] Swanson LM, Arnedt JT, Rosekind MR, Belenky G, Balkin TJ, Drake C (2011). Sleep disorders and work performance: findings from the 2008 national sleep foundation sleep in America poll. Journal of Sleep Research.

[ref-35] Tonetti L, Martoni M, Natale V (2011). Effects of different mattresses on sleep quality in healthy subjects: an actigraphic study. Biological Rhythm Research.

[ref-36] Van Dierendonck D, Te Nijenhuis J (2005). Flotation restricted environmental stimulation therapy (REST) as a stress-management tool: a meta-analysis. Psychology & Health.

[ref-37] Verhaert V (2011). Ergonomic analysis of integrated bed measurements: towards smart sleep systems.

[ref-38] Verhaert V, Druyts H, Van Deun D, De Wilde T, Van Brussel K, Haex B, Sloten JV (2012a). Modeling human-bed interaction: the predictive value of anthropometric models in choosing the correct bed support. Work.

[ref-39] Verhaert V, Druyts H, Van Deun D, Exadaktylos V, Verbraecken J, Vandekerckhove M, Haex B, Vander Sloten J (2012b). Estimating spine shape in lateral sleep positions using silhouette-derived body shape models. International Journal of Industrial Ergonomics.

[ref-52] Verhaert V, Haex B, Wilde TD, Berckmans D, Verbraecken J, Valck ED, Sloten JV (2011). Ergonomics in bed design: the effect of spinal alignment on sleep parameters. Ergonomics.

[ref-40] Verhaert V, Haex B, De Wilde T, Berckmans D, Vandekerckhove M, Verbraecken J, Sloten JV (2011a). Unobtrusive assessment of motor patterns during sleep based on mattress indentation measurements. IEEE Transactions on Information Technology in Biomedicine.

[ref-41] Verhaert V, Haex B, Wilde TD, Berckmans D, Verbraecken J, Valck Ed, Sloten JV (2011b). Ergonomics in bed design: the effect of spinal alignment on sleep parameters. Ergonomics.

[ref-42] Verhaert V, Van Deun D, Verbraecken J, Vandekerckhove M, Exadaktylos V, Haex B, Vander Sloten J (2013). Smart control of spinal alignment through active adjustment of mechanical bed properties during sleep. Journal of Ambient Intelligence and Smart Environments.

[ref-43] Wang Y, Wong DWC, Zhang M (2016). Computational models of the foot and ankle for pathomechanics and clinical applications: a review. Annals of Biomedical Engineering.

[ref-44] Wong DWC, Wang Y, Chen TLW, Leung AKL, Zhang M (2017). Biomechanical consequences of subtalar joint arthroereisis in treating posterior tibial tendon dysfunction: a theoretical analysis using finite element analysis. Computer Methods in Biomechanics and Biomedical Engineering.

[ref-45] Wong DWC, Wang Y, Leung ALK, Yang M, Zhang M (2018). Finite element simulation on posterior tibial tendinopathy: load transfer alteration and implications to the onset of pes planus. Clinical Biomechanics.

[ref-46] Wong DWC, Zhang M, Yu J, Leung ALK (2014). Biomechanics of first ray hypermobility: an investigation on joint force during walking using finite element analysis. Medical Engineering & Physics.

[ref-47] Woolf AD, Erwin J, March L (2012). The need to address the burden of musculoskeletal conditions. Best Practice & Research Clinical Rheumatology.

[ref-48] Wu J, Yuan H, Li X (2018). A novel method for comfort assessment in a supine sleep position using three-dimensional scanning technology. International Journal of Industrial Ergonomics.

[ref-49] Yoshida H, Kamijo M, Shimizu Y (2012). A study to investigate the sleeping comfort of mattress using finite element method. Kansei Engineering International Journal.

[ref-50] Zhang C, Zhang W, Webb DJ, Peng G-D (2010). Optical fibre temperature and humidity sensor. Electronics Letters.

[ref-51] Zhong S, Shen L, Zhou L, Guan Z (2014). Predict human body indentation lying on a spring mattress using a neural network approach. Proceedings of the Institution of Mechanical Engineers, Part H: Journal of Engineering in Medicine.

